# Familial MEN1 Syndrome Diagnosed on Functional Imaging: A Case Report with Clinical and Genetic Correlation

**DOI:** 10.1055/s-0043-1768448

**Published:** 2023-12-04

**Authors:** Ashwini Kalshetty, Ashwini Chalikandy

**Affiliations:** 1 Radiation Medicine Centre, Tata Memorial Hospital Annexe, BARC, Mumbai, Maharashtra, India; 2 Radiation Medicine Centre, Homi Bhabha National Institute, Mumbai, Maharashtra, India

**Keywords:** genetic, hyperparathyroidism, MEN1, NET, PET-CT, phenocopies

## Abstract

Multiple endocrine neoplasia, type 1 (MEN1) syndrome is an autosomal dominant disease characterized by tumors involving parathyroid, pituitary, and pancreas. The diagnosis is mostly clinical and by the presence of MEN1 gene mutation. We present a case with initial presentation of neuroendocrine tumor of pancreas whose ancillary findings on ^68^ Ga-DOTATATE positron emission tomography-computed tomography helped in raising suspicion of MEN1, which was confirmed on genetic testing and family history. We emphasize the importance of using gestalt approach in such cases to avoid misdiagnosis or delay. Additionally, we describe the clinical profile of affected family members with their MEN1 gene mutation status, highlighting the gestalt approach again to uncover the unknowns.

## Introduction

Multiple endocrine neoplasia, type 1 (MEN 1) syndrome is an autosomal dominant disease with a high degree of penetrance. It is caused by an inactivating mutation in tumor suppressor gene MEN 1 that encodes amino acid protein menin. 1 2 The prevalence of MEN 1 syndrome is estimated to be 2 to 10 people per 1,00,000. 3 4

MEN1 syndrome is characterized by occurrence of parathyroid, pituitary, and pancreatic tumors. Additionally, some patients develop adrenocortical tumors, meningioma, lipoma, face angiofibromas, and collagenomas, as well as pheochromocytomas, although rarely. 1 2 Primary hyperparathyroidism is the most common tumor in MEN 1 syndrome, occurring in approximately 90% of patients. Incidence of pancreatic neuroendocrine tumor (NET) in MEN1 syndrome varies from 30 to 80%. 1 More than 50% of all duodenopancreatic NETs in MEN1 patients are gastrinomas, while insulinomas constitute around 10 to 30%. 1

MEN1 syndrome is diagnosed if any of the three criteria are met: a patient with two or more MEN1-associated cancers, a patient with MEN1-associated tumor, and a first-degree relative who has MEN1, or a carrier of the MEN1 gene mutation. 1 5

Around 90% of the disease is inherited, while 8 to 10% occurs de novo. 1 2 The high frequency of phenocopies or, on the other hand, the difficulty in distinguishing between familial and sporadic instances due to early parental mortality, inadequate assessment of the family, and adoption might lead to a misdiagnosis of MEN1 syndrome. 5 6

Early detection of dormant cancers is now achievable due to the widespread use of cutting-edge imaging techniques. ^68^ Ga-DOTATATE PET-CT (positron emission tomography-computed tomography) is frequently used in the management of NETs. In this article, we present a case of familial MEN1 syndrome that was discovered using a Gestalt analysis of PET-CT observations along with genetic test results of index case and his family members.

## Case Findings

**Fig. 1:**
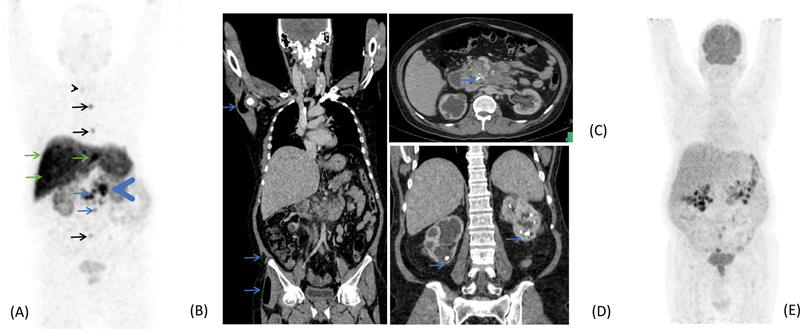
^68^ Ga-DOTATATE PET-CT (positron emission tomography-computed tomography). ( **A** ) Maximum intensity projection (MIP) showing multiple somatostatin receptor expressing lesions in pancreatic tail ( *blue arrowhead* ), abdominal nodes ( *blue arrows* ), multiple hepatic deposits ( *green arrows* ), sclerotic skeletal lesions ( *black arrows* ), and focal uptake in tracheoesophageal groove ( *black arrowhead* ). ( **B** ) Multiple intramuscular lipomas ( *blue arrows* ). ( **C** ) Calcification in head and uncinate process of pancreas ( *blue arrow* ). ( **D** ) Bilateral nephrolithiasis ( *blue arrows* ). ( **E** ) MIP of fluorodeoxyglucose PET-CT showing insignificant metabolic activity in the lesions.

^68^ Ga-DOTATATE PET-CT done in our center showed somatostatin receptor (SSTR) expressing hypodense lesions involving uncinate process and tail of pancreas, multiple hypodense liver lesions, and sclerotic skeletal lesions. There was well-defined lung nodule measuring 1.2 × 2 cm in the right lung lower lobe which did not show any significant tracer uptake, suspicious of metastases. Other findings noted were multiple bilateral renal calculi causing hydronephrosis and multiple intramuscular lipomas in left gluteal, left abdominal wall, left teres major, left infraspinatus, and other smaller ones in thigh muscles. There were specks of calcification in and around pancreatic region that was initially attributed to sequelae of alcoholic pancreatitis. There were surgical staples in neck (mostly around left lobe of thyroid) with an ill-defined hyperdense lesion near its lower pole. There were ill-defined foci of increased tracer in bilateral tracheoesophageal grooves and near lower pole of right lobe of thyroid. Fluorodeoxyglucose (FDG) PET-CT showed no significant tracer uptake in any of the lesions.

A 56-year-old male with metastatic duodenal (NET) was referred to our clinic for additional peptide receptor radionuclide therapy (PRRT). He was diagnosed to have type II diabetes (on oral hypoglycemics), chronic pancreatitis due to alcohol, and history of laparoscopic cholecystectomy for gall stones. He was diagnosed with NET 6 months back and was started on sunitinib and zoledronic acid for 2 months. He had to discontinue the medications due to side effects and financial reasons. He was the started on monthly inj. octreotide long-acting release (LAR) 30 mg for 5 months. He underwent CT angiography that showed multiple hypervascular hepatic lesions and extensive necrotic tumor 5.2 × 2.3 × 4 cm with minimal hypervascularity in duodenum, involving adjacent mesentery with near occlusive tumor thrombus in splenic vein, superior mesenteric vein, and multiple necrotic nodes. He was then advised PRRT from a different hospital where he received 2 cycles of PRRT (cumulative dose: 378 mCi of ^177^ Lu-DOTATATE). We reviewed his baseline ^68^ Ga-DOTATATE PET-CT and current ^68^ Ga-DOTATATE PET-CT at the time of evaluation and noted the findings ( Fig. 1 ).

**Fig. 2:**
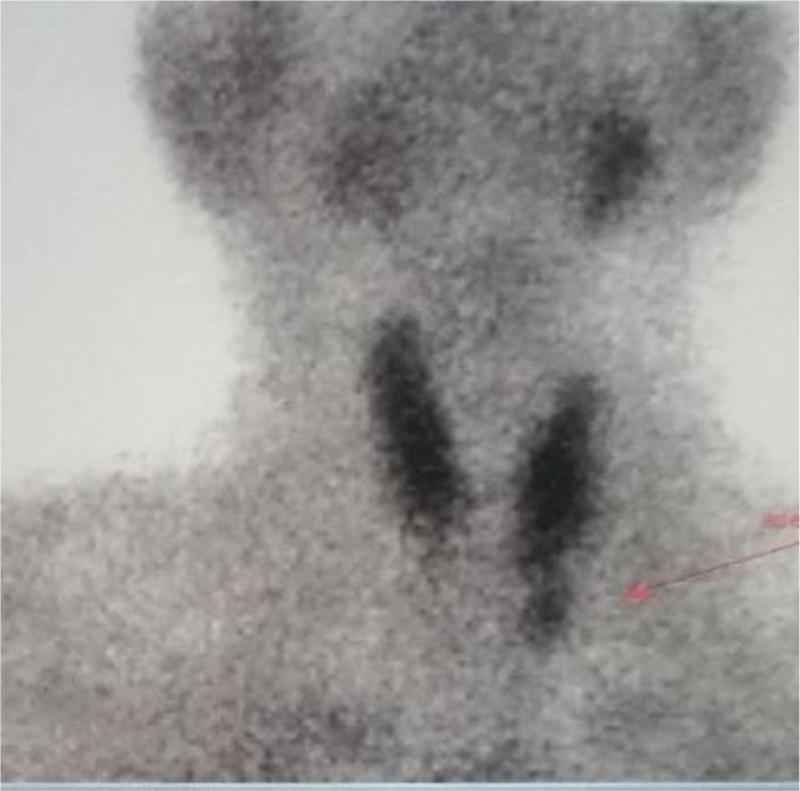
^99^ m-Tc sestamibi parathyroid scan of index case at the age of 51 years showing recurrent left inferior parathyroid adenoma ( *arrow* ).

Noting these ancillary findings, additional history was elicited from him and his relatives. He then gave history of recurrent renal calculi, acute pancreatitis, and vertigo when he was 48 years old. His old documents mentioned left inferior parathyroid adenoma and he underwent left inferior parathyroidectomy. He presented to emergency department 3 years later with abdominal pain and vomiting. Ultrasonography (USG) abdomen showed bilateral medullary nephrocalcinosis, right renal and ureteric calculi with right hydroureteronephrosis. His serum primary hyperparathyroidism (PTH) was raised (180 pg/mL) with serum calcium of 10.3 mg/dL. He was suspected of recurrent hyperparathyroidism and was confirmed to have recurrent left inferior parathyroid adenoma on ^99^ mTc-sestamibi parathyroid scan ( Fig. 2 ). USG neck for parathyroid corroborated by showing a well-defined ovoid hypoechoic nodule measuring 7 × 4 mm seen located inferior to lower pole of left thyroid gland, suggestive of left inferior parathyroid nodule. He underwent left inferior parathyroidectomy again and remained asymptomatic for 4 years. However, he then complained of recurrence of abdominal pain. CT abdomen showed multiple hypervascular hepatic lesions, hypervascular lesion in uncinate, duodenal wall, and extensive lymphadenopathy. Endoscopic ultrasound-guided fine-needle aspiration cytology of paraduodenal lymph node was suggestive of NET grade II, synaptophysin, and chromogranin positive with Ki67 index of 5 to 10%. Serum chromogranin A (CgA) was raised (373 ng/mL).

His son revealed h/o insulinoma at the age of 25 years and had undergone exploratory laparotomy with spleen preserving distal pancreatectomy. He was being monitored annually in another hospital and is under remission for 5 years now. Patient's father succumbed to a suspicious gastric malignancy with metastases on abdominal contrast-enhanced CT. He did not undergo any specific medical care and his diagnosis was never confirmed on histopathology. His second daughter had multiple joint pains and was being investigated elsewhere. Her serum PTH found to be raised (536 pg/mL) and ^99^ mTc-parathyroid scan showed left inferior parathyroid adenoma.

Based on the clinical findings and family history, familial MEN 1 syndrome was considered and genetic counselling for family was advised.

**Fig. 3:**
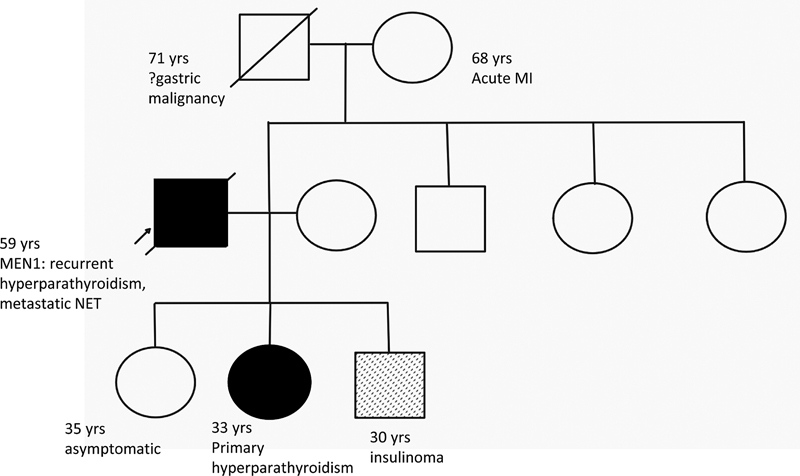
Pedigree chart showing the MEN-1 inheritance pattern in family members with their clinical profile.

The genetic test for MEN1 gene mutation was done for the family and he was found to harbor MEN 1 gene mutation in Exon 10 of chromosome11q13 during segregation analysis. The results of other family members with their clinical profiles are summarized in ( Fig. 3 ).

**Fig. 4:**
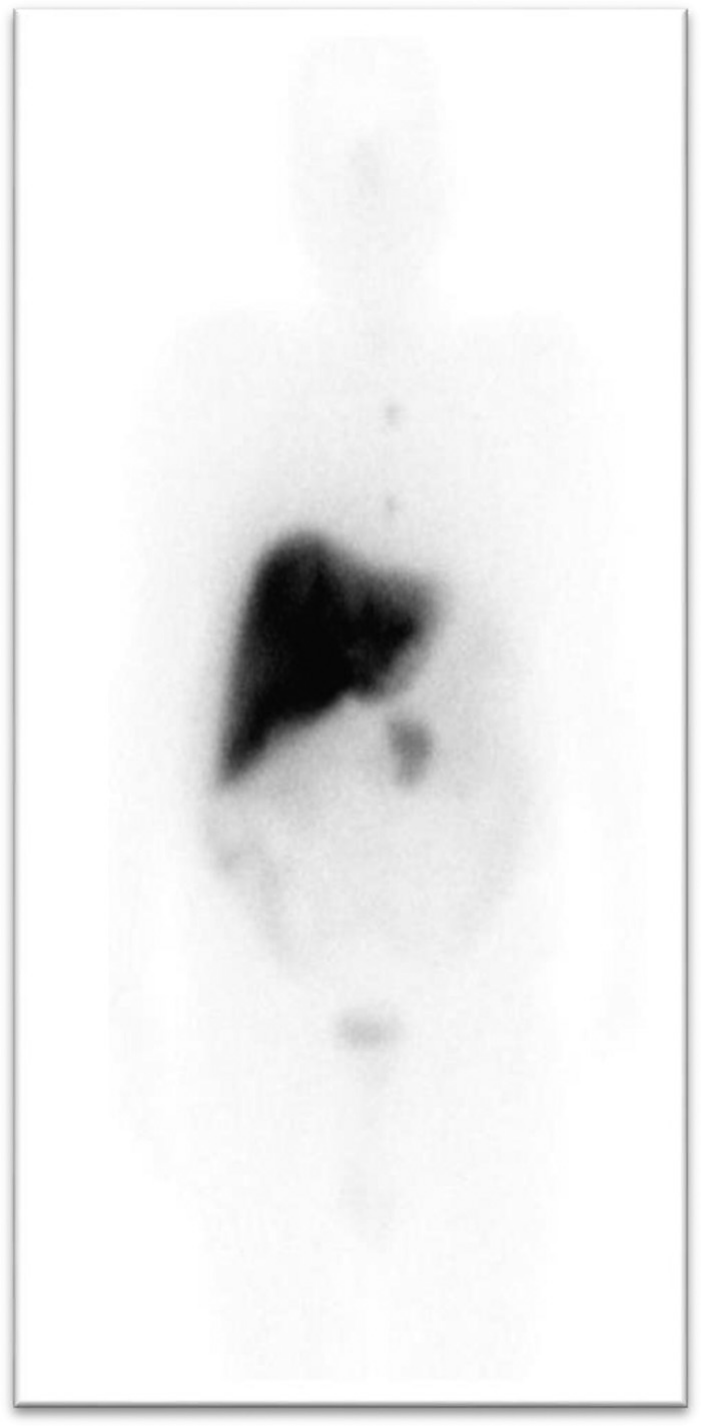
Post-therapy scan showing concordant accumulation of ^177^ Lu-DOTATATE at tumor sites.

**Fig. 5:**
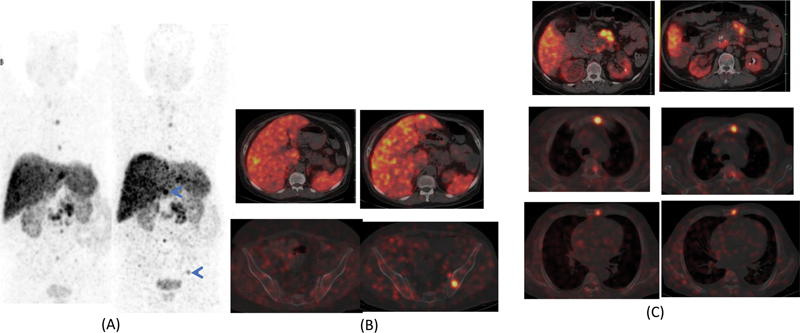
( **A** ) Comparative maximum intensity projection of ^68^ Ga-DOTATATE positron emission tomography-computed tomography showing new somatostatin expressing hepatic and skeletal lesions ( *blue arrows* ). ( **B** ) Axial fused images demonstrating new hepatic and skeletal lesions ( *on the right* ) showing somatostatin receptor overexpression. ( **C** ) The pancreatic and duodenal lesions, peripancreatic lymphadenopathy, and skeletal lesions showing stable to partial response ( *on the right* ).

He was continued on PRRT for the inoperable primary NET and metastatic disease ( Fig. 4 ). He received 5# PRRT (cumulative dose of 924 mCi ^177^ Lu-DOTATATE). However, he then complained of increased frequency of loose motions with high Sr. CgA; corroborating with disease progression ( Fig. 5 ). He was referred to medical oncologist for restarting octreotide and tyrosine kinase inhibitors. However, he went never consulted the medical Oncologist later. He was hospitalized in a local hospital with generalized weakness and abdominal distention 10 months later. He developed ascites, bilateral pleural effusion, and pericardial effusion. He underwent ascitic taping and was put on conservative management. However, within a month he was again admitted for abdominal distension, loose motions, shortness of breath, and loss of appetite. His white blood cell counts were raised (14,000/mm ^3^ ); serum creatinine was marginally increased (1.9 mg/dL), serum albumin was in low range (2.17 g/dL), and serum alkaline phosphatase raised (716 U/L). He was started on intravenous antibiotics and underwent large volume paracentesis under albumin cover; fluid cytology was negative for malignancy. We later learnt that he had succumbed 20 days after developing sudden onset breathlessness. We could not get other details and exact cause of death.

## Discussion

The availability of advanced imaging technologies has the potential to provide additional and timely information, but warranting its appropriate use in patients. This case study emphasizes the value of taking a comprehensive approach when new observations are made. The index case had consulted several hospitals for various symptoms over the course of 8 to 9 years. The combination of patient's limited disclosure of information and other hospitals' use of a symptom-focused approach led to delayed diagnosis in these circumstances. Additionally, this caused the genetic testing of family members who were receiving treatment elsewhere to be delayed. Awareness of the spectrum of clinical manifestations like recurrent hyperparathyroidism that presented as recurrent renal calculi, pancreatitis on imaging, and multiple lipomas helped in raising the suspicion for MEN1 syndrome.

Another important point is frequent association of phenocopies in family members of MEN1. 7 Any gastro-entero-pancreatic (GEP)-NET occurrence in people under 30 years of age was highly predictive of MEN1 mutation, but this was not the case for the patient's son ( Fig. 3 ). This observation has raised numerous questions about them being true phenocopies, genotype-phenotype correlations, and possibility of other mutations. 1 7 Additional testing for CDKN1B mutation may be recommended in phenocopies. 1

Unfavorable clinical outcomes may result from the MEN 1 syndrome's aggressive phenotypic presentation. These include parathyroid carcinoma, malignant insulinoma and glucagonoma, pituitary carcinoma, adrenocortical carcinoma, ovarian NETs, and nonendocrine malignancies including breast cancer, hepatocellular carcinoma, melanoma, lung adenocarcinoma, renal cell carcinoma, papillary thyroid cancer, and prostate cancer. 8 Therefore, a holistic approach for family members of affected individuals is essential; especially using whole body imaging to identify dormant tumors or lesions. This approach should be adopted particularly for the second daughter who has MEN1 gene mutation, but has only shown hyperparathyroidism so far, requiring active surveillance for the development of other symptoms as per the guidelines. 1 Adequate, targeted long-term surveillance and multidisciplinary approach with comprehensive documentation can reduce morbidity and mortality effectively.

The genotype–phenotype relationships are not conclusively demonstrable requiring need for multidisciplinary surveillance especially for the aggressive disease. 8 This also showcases the gaps in our knowledge of the etiopathogenesis of this syndrome. Large clinical trials are difficult due to MEN1 being a relatively rare disease. Therefore, comprehensive contribution to rare disease registries and repositories is essential to augment research in this domain.

## Sensitivity of [68]GA-DOTATATE PET-CT in GEP-NETs

There is ample evidence of the usefulness of ^68^ GA-DOTATATE PET-CT in MEN1 syndrome showing higher sensitivity in detecting lesions, mostly NETs. 9 10 11 However, occurrences of benign pathologies like lipomas, renal calculi, and pancreatic calcification like in our case are usually overlooked. These are important pointers and may not always appear as hot areas on molecular imaging. Also, it would be crucial to incorporate ^68^ GA-DOTATATE PET-CT imaging in new practice standards considering the increasing volume of recent research supporting its value.

## Conclusion

The increasing use of advanced molecular imaging technologies like PET-CT and functional MRI have readily made detailed evaluation possible. Awareness and a comprehensive analytical approach with additional observations can help in identifying rare diseases like MEN1 syndrome. There is a need to distinguish true phenocopies from apparent ones to unlock the biological pathways, which warrants considering all the findings rather than just the clinical presentation and gene mutation analysis.

## Key Takeaways

- The ancillary findings on SSTR imaging must be reported and emphasized in a case of GEP-NET
- The high incidence of phenocopies related to MEN1 syndrome suggests restricted causal mechanisms of MEN1 gene mutation only. Additional testing for CDKN1B gene mutation and a holistic approach especially using advanced molecular imaging techniques will resolve the confounding nature of the disease.
- It is necessary to make a comprehensive contribution to rare disease repositories to advance research and understand the etiopathogenesis of this disease.

## References

[1] Endocrine Society Thakker R V Newey P J Walls G V Clinical practice guidelines for multiple endocrine neoplasia type 1 (MEN1) J Clin Endocrinol Metab 2012 97 09 2990 3011 22723327 10.1210/jc.2012-1230

[2] Kamilaris C DC Stratakis C A Multiple endocrine neoplasia type 1 (MEN1): an update and the significance of early genetic and clinical diagnosis Front Endocrinol (Lausanne) 2019 10 339 10.3389/fendo.2019.00339 31263451 PMC6584804

[3] Yoshimoto K Multiple endocrine neoplasia type 1: from bedside to benchside J Med Invest 2000 47(3-4):108 117 11019489

[4] Tepede A A Welch J Lee M 18F-FDOPA PET/CT accurately identifies MEN1-associated pheochromocytoma Endocrinol Diabetes Metab Case Rep 2020 2020 19 156; Epub ahead of print 10.1530/EDM-19-0156 PMC7077596 32130200

[5] Turner J J Christie P T Pearce S H Turnpenny P D Thakker R V Diagnostic challenges due to phenocopies: lessons from multiple endocrine neoplasia type1 (MEN1) Hum Mutat 2010 31 01 E1089 E1101 19953642 10.1002/humu.21170

[6] Falchetti A Genetics of multiple endocrine neoplasia type 1 syndrome: what's new and what's old. F1000Res. 2017 6 F1000 10.12688/f1000research.7230.1. doi: Faculty Rev-73. PMID: 28184288; PMCID: PMC5288685. PMC5288685 28184288

[7] Kövesdi A Tóth M Butz H True MEN1 or phenocopy? Evidence for geno-phenotypic correlations in MEN1 syndrome Endocrine 2019 65 02 451 459 31044390 10.1007/s12020-019-01932-x PMC6656790

[8] Mele C Mencarelli M Caputo M Phenotypes associated with MEN1 syndrome: a focus on genotype-phenotype correlations Front Endocrinol (Lausanne) 2020 11 591501 10.3389/fendo.2020.591501 33312161 PMC7708377

[9] Morgat C Vélayoudom-Céphise F L Schwartz P Evaluation of (68)Ga-DOTA-TOC PET/CT for the detection of duodenopancreatic neuroendocrine tumors in patients with MEN1 Eur J Nucl Med Mol Imaging 2016 43 07 1258 1266 26819103 10.1007/s00259-016-3319-3

[10] Sadowski S M Millo C Cottle-Delisle C Results of (68)Gallium-DOTATATE PET/CT scanning in patients with multiple endocrine neoplasia type 1 J Am Coll Surg 2015 221 02 509 517 26206648 10.1016/j.jamcollsurg.2015.04.005 PMC4515773

[11] Lastoria S Marciello F Faggiano A Role of (68)Ga-DOTATATE PET/CT in patients with multiple endocrine neoplasia type 1 (MEN1) Endocrine 2016 52 03 488 494 26242621 10.1007/s12020-015-0702-y

